
JOURNAL INFORMATION
==============================
NLM Title Abbreviation: J Diabetes Investig
Journal ID: J Diabetes Investig
NLM Journal ID: 101520702
Journal ID: JDI

Journal of Diabetes Investigation

ISSN: 2040-1116
EISSN: 2040-1124
Publisher: Wiley

ARTICLE INFORMATION
==============================
PMCID: PMC11474747
DOI: 10.1111/jdi.13853
Article ID: JDI13853
Article version: 1
Subjects: Issue Information, Issue Information

Issue Information

Electronic publication date: 2023 Jun 22
Publication date: 2023 Jul
Volume: 14
Issue: 7
First page: 825
Last page: 826
Copyright: © 2023 The Authors. Journal of Diabetes Investigation published by Asian Association for the Study of Diabetes (AASD) and John Wiley & Sons Australia, Ltd
License: This is an open access article under the terms of the http://creativecommons.org/licenses/by/4.0/ License, which permits use, distribution and reproduction in any medium, provided the original work is properly cited.
License URL: https://creativecommons.org/licenses/by/4.0/

Figure count: 0
Table count: 0
Page count: 5

source-schema-version-number: 2.0
cover-date: July 2023
details-of-publishers-convertor: Converter:WILEY_ML3GV2_TO_JATSPMC version:6.4.9 mode:remove_FC converted:15.10.2024

==============================

### JDI

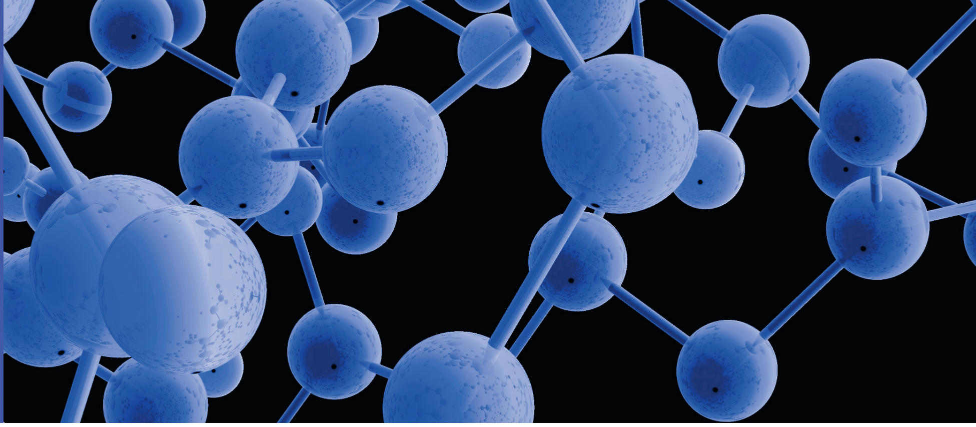

Volume 14 | Issue 7
July 2023

**Journal of Diabetes Investigation**
Official Journal of the Asian Association for the Study of Diabetes

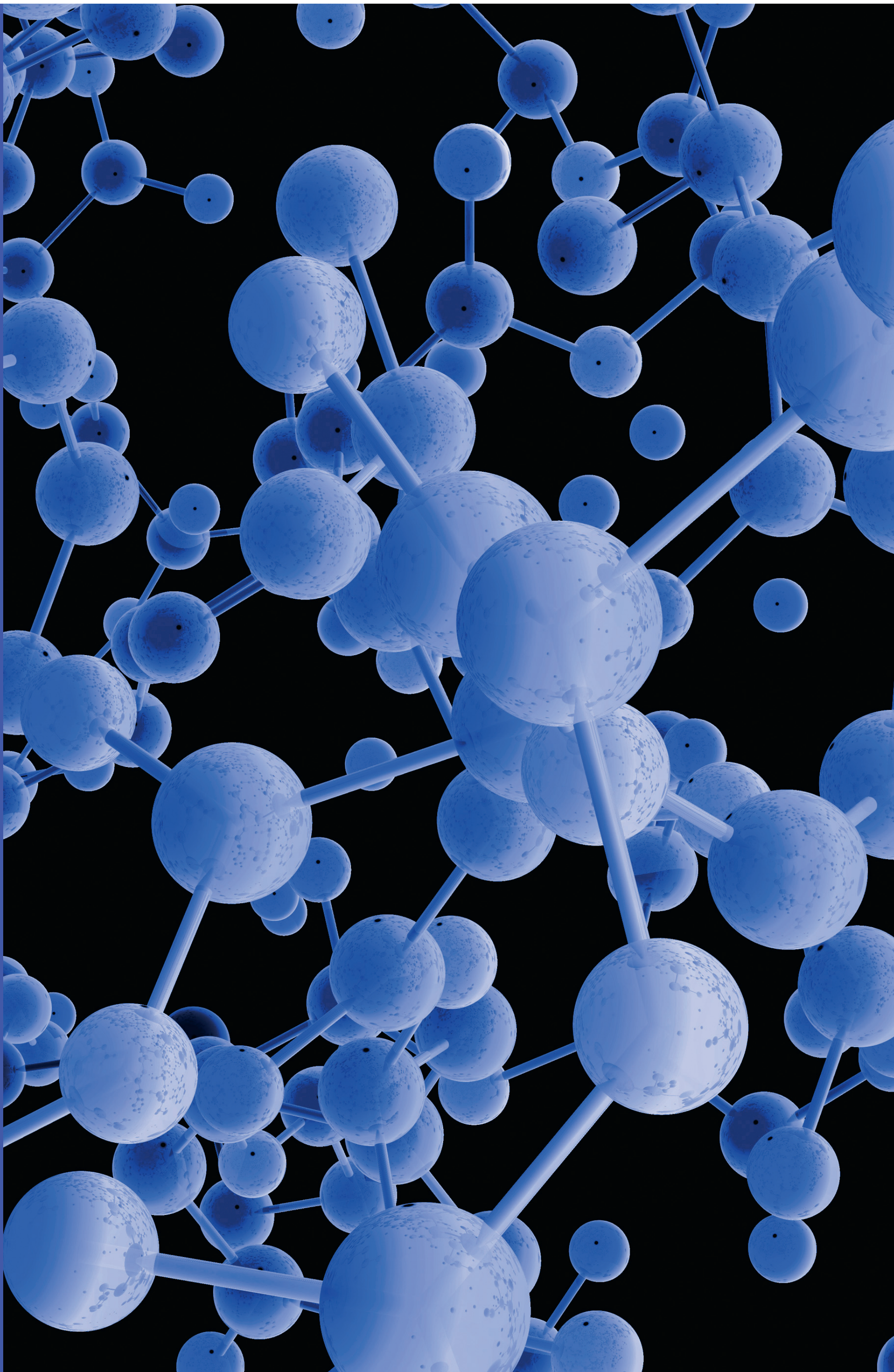

FFA vs TSC for reducing non-attendance

THE ASIAN ASSOCIATION FOR THE STUDY OF DIABETES
AASD
WILEY

### JDI Journal of Diabetes Investigation

Open access

Official Journal of the Asian Association for the Study of Diabetes

**Editor-in-Chief**

***Nigishi Hotta,*** *Japan Organization of Occupational Health and Safety, Chubu Rosai Hospital, Nagoya, Japan*

**Co-Editor**

***Domenico Accili,*** *Columbia University, New York, USA*

**Expert Editor**

***Bo Ahren,*** *Lund, Sweden*
***Young Min Cho,*** *Seoul, Korea*
***Sean Dinneen,*** *Galway, Ireland*
***Marc Gilbert,*** *Montpelier, France*
***Limei Liu,*** *Shanghai, China*
***Ronald Ma,*** *Hong Kong, China*
***Louis Philipson,*** *Chicago, USA*
***Wayne H-H Sheu,*** *Taichung, Taiwan*
***Bernard Thorens,*** *Lausanne, Switzerland*
***Daisuke Yabe,*** *Gifu, Japan*

**Editorial Board**

***Eiichi Araki,*** *Kumamoto, Japan*
***Xiaoping Chen,*** *Beijing, China*
***Lee-Ming Chuang,*** *Taipei, Taiwan*
***Masakazu Haneda,*** *Asahikawa, Japan*
***Larry Low-Tone Ho,*** *Taipei, Taiwan*
***Tianpei Hong,*** *Beijing, China*
***Akihisa Imagawa,*** *Osaka, Japan*
***Nobuya Inagaki,*** *Kyoto, Japan*
***Linong Ji,*** *Beijing, China*
***Weiping Jia,*** *Shanghai, China*
***Yi-Der Jiang,*** *Taipei, Taiwan*
***Takashi Kadowaki,*** *Tokyo, Japan*
***Kohei Kaku,*** *Kurashiki, Japan*
***Keizo Kanasaki,*** *Izumo, Japan*
***Doo-Man Kim,*** *Seoul, Korea*
***Karen Lam,*** *Hong Kong, China*
***Hong Kyu Lee,*** *Gyeonggi-do, Korea*
***In-Kyu Lee,*** *Daegu, Korea*
***Moon-Kyu Lee,*** *Seoul, Korea*
***Jiro Nakamura,*** *Nagakute, Japan*
***Kishio Nanjo,*** *Wakayama, Japan*
***Wataru Ogawa,*** *Kobe, Japan*
***Yutaka Seino,*** *Osaka, Japan*
***Shyi-Jang Shin,*** *Kaohsiung, Taiwan*
***Hirohito Sone,*** *Niigata, Japan*
***Kathryn Tan,*** *Hong Kong, China*
***Yukio Tanizawa,*** *Ube, Japan*
***Kohjiro Ueki,*** *Tokyo, Japan*
***Jun Wada,*** *Okayama, Japan*
***Hirotaka Watada,*** *Tokyo, Japan*
***Jianping Weng,*** *Guangzhou, China*
***Soroku Yagihashi,*** *Hirosaki, Japan*
***Hiroshi Yamamoto,*** *Kanazawa, Japan*
***Toshimasa Yamauchi,*** *Tokyo, Japan*
***Wenying Yang,*** *Beijing, China*
***Kazuki Yasuda,*** *Tokyo, Japan*
***Koutaro Yokote,*** *Chiba, Japan*
***Kun-Ho Yoon,*** *Seoul, Korea*
***Dalong Zhu,*** *Nanjing, China*

**International Editorial Board**

***Michael A. Brownlee,*** *New York, USA*
***Subrata Chakrabarti,*** *Ontario, Canada*
***Mark E. Cooper,*** *Prahran, Australia*
***Philippe Froguel,*** *London, UK*
***Wilfred Fujimoto,*** *Hawaii, USA*
***Michael S. German,*** *San Francisco, USA*
***Andrew Hattersley,*** *Exeter, UK*
***Jean-Claude Henquin,*** *Brussels, Belgium*
***Jens Juul Holst,*** *Copenhagen, Denmark*
***Frank B. Hu,*** *Boston, USA*
***C. Ronald Kahn,*** *Boston, USA*
***George L. King,*** *Boston, USA*
***Rayaz A. Malik,*** *Manchester, UK*
***Michael Mauer,*** *Minneapolis, USA*
***Carl Erik Mogensen,*** *Aarhus, Denmark*
***Steven E. Shoelson,*** *Boston, USA*
***Jay S. Skyler,*** *Miami, USA*
***Paul Zimmet,*** *Melbourne, Australia*

**Assistant Editorial Board**

***Tien-Jyun Chang,*** *Taipei, Taiwan*
***Yoshiyuki Hamamoto,*** *Osaka, Japan*
***Norio Harada,*** *Kyoto, Japan*
***Yi-Jen Hung,*** *Taipei, Taiwan*
***Chi-Min Hwu,*** *Taipei, Taiwan*
***Hideki Kamiya,*** *Aichi, Japan*
***Dae Jung Kim,*** *Suwon, Korea*
***Seung-Hwan Lee,*** *Seoul, Korea*
***Hung-Yuan Li,*** *Taipei, Taiwan*
***Takashi Matsuzaka,*** *Tsukuba, Japan*
***Keiko Naruse,*** *Aichi, Japan*
***Takeshi Nishikawa,*** *Kumamoto, Japan*
***Eung-Jung Rhee,*** *Seoul, Korea*
***Yusuke Seino,*** *Aichi, Japan*
***Hironori Waki,*** *Tokyo, Japan*

**Aims and Scope:** *Journal of Diabetes Investigation* is your core diabetes journal from Asia; the official journal of the Asian Association for the Study of Diabetes (AASD). The journal publishes original research, country reports, commentaries, reviews, mini-reviews, case reports, letters, as well as editorials and news. The journal embraces clinical and experimental research in diabetes and related areas. This includes aspects of prevention, treatment, as well as molecular aspects and pathophysiology. Translational research focused on the exchange of ideas between clinicians and researchers is also welcome.

**Address for Editorial Correspondence:**
The Editor, *Journal of Diabetes Investigation*, Nomura Fudosan Nishi Shinjuku Building, 8F 8-4-2 Nishi Shinjuku, Shinjuku-ku, Tokyo 160-0023, Japan. Tel: +81 3 4520 9009; Fax: +81 3 4520-9059 Email: JDI@wiley.com

**Copyright © 2023 Asian Association for the Study of Diabetes and John Wiley & Sons Australia, Ltd**

**Cover:** Insulin molecule © Pasieka/Science Photo Library

This journal is available online at Wiley Online Library. Visit wileyonlinelibrary.com to search the articles and register for table of contents and e-mail alerts.

**For submission instructions, and all other information visit wileyonlinelibrary.com/journal/jdi**

ISSN 2040-1124 (Online)

JDI.JEB.Jul23

WILEY

**Publisher**
*Journal of Diabetes Investigation* is published by John Wiley & Sons Australia, Ltd
Level 19, 15 William Street,
Melbourne, Victoria 3000,
Australia.
Tel: +61 (0)3 9274 3100

**Production Editor**
Jayalakshmi Mohan (email: JDIproduction@wiley.com)

**Abstracting and Indexing Services**
The Journal is indexed by Academic OneFile (GALE Cengage), Biological Abstracts (Clarivate Analytics), Biological Science Database (ProQuest), BIOSIS Previews (Clarivate Analytics), Embase (Elsevier), Health & Medical Collection (ProQuest), Health Research Premium Collection (ProQuest), Hospital Premium Collection (ProQuest), MEDLINE/PubMed (NLM), Natural Science Collection (ProQuest), ProQuest Central (ProQuest), Science Citation Index Expanded (Clarivate Analytics), SciTech Premium Collection (ProQuest), SCOPUS (Elsevier), Web of Science (Clarivate Analytics).

**Open Access and Copyright**
All articles published by *Journal of Diabetes Investigation* are fully open access: immediately freely available to read, download and share. All *Journal of Diabetes Investigation* articles accepted from 24 September 2013 are published under the terms of the Creative Commons Attribution 4.0 Unported (CC BY 4.0) license, which permits use, distribution and reproduction in any medium, provided the original work is properly cited. Articles accepted before this date were published under the agreement as stated in the final article. Copyright on any *Journal of Diabetes Investigation* article accepted after 24 September 2013, the date on which *Journal of Diabetes Investigation* became an open access journal, is retained by the author(s). Authors grant to *Journal of Diabetes Investigation* and Wiley a license to publish the article and for Wiley to identify itself as the original publisher. Authors also grant any third party the right to use the article freely as long as its integrity is maintained and its original authors, citation details and publisher are identified. Please visit https://authorservices.wiley.com/author-resources/Journal-Authors/open-access/hybrid-open-access.html for further information about Open Access.

**Use by commercial ''for-profit'' organisations (not applicable to CC BY 4.0 licensed articles)**
Use of Wiley Open Access articles for commercial, promotional, or marketing purposes requires further explicit permission from Wiley and will be subject to a fee. Requests to reproduce material from this journal content are being handled through the Rightslink® online service.

- Locate the article you wish to reproduce on Wiley Online Library (http://onlinelibrary.wiley.com)
- Navigate to the abstract page
- Click on the 'Request Permissions' link, under the 'ARTICLE TOOLS' menu
- Follow the online instructions and select your requirements from the drop down options and click on 'quick price' to get a quote
- Create a RightsLink® account to complete your transaction (and pay, where applicable)
- Read and accept our Terms & Conditions and Download your license
- For any technical queries please contact customercare@copyright.com
- For further information and to view a Rightslink® demo please visit www.wiley.com and select Rights & Permissions.
- Print reprints of Wiley Open Access articles can be purchased from corporatesales@wiley.com.

**Disclaimer**
The Publisher, the Asian Association for the Study of Diabetes, and Editors cannot be held responsible for errors or any consequences arising from the use of information contained in this journal; the views and opinions expressed do not necessarily reflect those of the Publisher, the Asian Association for the Study of Diabetes, or Editors, neither does the publication of advertisements constitute any endorsement by the Publisher, the Asian Association for the Study of Diabetes, or Editors of the products advertised.

Wiley Open Access articles posted to repositories or websites are without warranty from Wiley of any kind, either express or implied, including, but not limited to, warranties of merchantability, fitness for a particular purpose, or noninfringement.
To the fullest extent permitted by law Wiley disclaims all liability for any loss or damage arising out of, or in connection, with the use of or inability to use the content.

**Journal Customer Services:** For ordering information, claims and any enquiry concerning your journal subscription please go to https://wolsupport.wiley.com/s/contactsupport?tabset-a7d10=2 or contact your nearest office.
**Americas:** Email: cs-journals@wiley.com; Tel: +1 877 762 2974
**Europe, Middle East and Africa:** Email: cs-journals@wiley.com; Tel: +44 (0) 1865 778315; 0800 1800 536 (Germany)
**Germany, Austria, Switzerland, Luxembourg, Liechtenstein:** cs-germany@wiley.com; Tel: 0800 1800 536 (Germany)
**Asia Pacific:** Email: cs-journals@wiley.com; Tel: +65 3165 0890.
**Japan:** For Japanese speaking support, Email: cs-japan@wiley.com; .
**Visit our Online Customer Help** at https://wolsupport.wiley.com/s/contactsupport?tabset-a7d10=2

Wiley's Corporate Citizenship initiative seeks to address the environmental, social, economic, and ethical challenges faced in our business and which are important to our diverse stakeholder groups. Since launching the initiative, we have focused on sharing our content with those in need, enhancing community philanthropy, reducing our carbon impact, creating global guidelines and best practices for paper use, establishing a vendor code of ethics, and engaging our colleagues and other stakeholders in our efforts. Follow our progress at www.wiley.com/go/citizenship

VOLUME 14 – ISSUE 7 – JULY 2023

### Journal of Diabetes Investigation

Open access

### Review

**Newly discovered knowledge pertaining to glucagon and its clinical applications**
Dan Kawamori and Shugo Sasaki **829**

### Commentary

**Gut as a source of new β-cells and Segi's cap**
Soroku Yagihashi **838**

### Update

**The era of continuous glucose monitoring and its expanded role in type 2 diabetes**
Jin Yu, Jae-Hyoung Cho, and Seung-Hwan Lee **841**

### Original Articles

### Basic Science and Research

**Linagliptin ameliorated cardiac fibrosis and restored cardiomyocyte structure in diabetic mice associated with the suppression of necroptosis**
Juthi Adhikari, Taro Hirai, Emi Kawakita, Kunimitsu Iwai, Daisuke Koya, and Keizo Kanasaki **844**

### Clinical Science and Care

**Comparison of positive rates between glutamic acid decarboxylase antibodies and ElisaRSR™ 3 Screen ICA™ in recently obtained sera from patients who had been previously diagnosed with slowly progressive type 1 diabetes**
Nobuaki Takehana, Tomoyasu Fukui, Yusaku Mori, Munenori Hiromura, Michishige Terasaki, Makoto Ohara, Michiya Takada, Masako Tomoyasu, Yoshihisa Ito, Tetsuro Kobayashi, and Sho-ichi Yamagishi **856**

**Cognitive protection of incretin-based therapies in patients with type 2 diabetes mellitus: A systematic review and meta-analysis based on clinical studies**
Sanbao Chai, Fengqi Liu, Shuqing Yu, Zhirong Yang, and Feng Sun **864**

**Independent risk factors of rapid glomerular filtration rate decline in patients with type 2 diabetes with preserved kidney function and normoalbuminuria: A multicenter cohort study**
Daishi Hirano, Hiroyuki Unoki-Kubota, Toshiyuki Imasawa, Ritsuko Yamamoto-Honda, Hiroshi Kajio, Shigeo Yamashita, Yuka Fukazawa, Naoto Seki, Mitsuhiko Noda, and Yasushi Kaburagi **874**

**Patient referral flow between physician and ophthalmologist visits for diabetic retinopathy screening among Japanese patients with diabetes: A retrospective cross-sectional cohort study using the National Database**
Noriko Ihana-Sugiyama, Takehiro Sugiyama, Takao Hirano, Kenjiro Imai, Mitsuru Ohsugi, Ryo Kawasaki, Toshinori Murata, Youichiro Ogura, Kohjirou Ueki, Toshimasa Yamauchi, and Takashi Kadowaki **883**

**Validation and application of the 2019 International Working Group on the Diabetic Foot risk stratification for diabetic foot in Chinese patients**
Xiling Hu, Yao Zhang, Yanming Chen, and Yin-Ping Zhang **893**

### Short Report

**The influence of triglycerides on small dense low-density lipoprotein cholesterol levels is attenuated in low low-density lipoprotein-cholesterol range: Implications for the negative results of the PROMINENT trial**
Tsutomu Hirano and Yasuki Ito **902**

### Original Article

**Behavioral change stage might moderate the impact of multifaceted interventions on non-attendance from medical care among patients with type 2 diabetes: The Japan Diabetes Outcome Intervention Trial-2 Large-Scale Trial 007 ( J-DOIT2-LT007 )**
Ryotaro Bouchi, Mitsuhiko Noda, Yasuaki Hayashino, Atsushi Goto, Katsuya Yamazaki, Hikari Suzuki, Toshiaki A Furukawa, Kazuo Izumi, Masashi Kobayashi, and J-DOIT2 Study Group **907**

### Case Report

**Diabetes technology including automated insulin delivery systems to manage hyperglycemia in a failing pancreatic graft: Case series of people with type 1 diabetes and a pancreas kidney or pancreas-only transplant**
Dimitra Stathi, Thomas Johnston, Rebecca Hyslop, Anna Brackenridge, and Janaka Karalliedde **917**
